# Evaluation of cowpea (*Vigna unguiculata* (L.) Walp.) landraces to bacterial blight caused by *Xanthomonas axonopodis* pv. *vignicola*

**DOI:** 10.1016/j.cropro.2018.10.013

**Published:** 2019-02

**Authors:** Hammed A. Durojaye, Yonnelle D. Moukoumbi, Victor O. Dania, Ousmane Boukar, Ranajit Bandyopadhyay, Alejandro Ortega-Beltran

**Affiliations:** a Department of Crop Protection and Environmental Biology, University of Ibadan, Nigeria; b International Institute of Tropical Agriculture (IITA), PMB 5320, Ibadan, Nigeria; c IITA, Kano Station, Nigeria; d National Institute of Agricultural Research, Gros Bouquet, PMB 16 169, Libreville, Gabon

**Keywords:** Disease resistance, Traditional landraces, Bacterial blight

## Abstract

Cowpea is an important protein source for human populations in many nations across sub-Saharan Africa (SSA). However, cowpea production is constrained by bacterial blight (CoBB) caused by *Xanthomonas axonopodis* pv. *vignicola* (*Xav*), a disease affecting most cowpea-growing areas. A large proportion of smallholder farmers across SSA rely on traditional cowpea landraces (CLR) to produce the crop. The International Institute of Tropical Agriculture (IITA) possesses the largest collection of cowpea germplasm, including several CLR accessions. However, screening for resistance to CoBB in most of the CLR accessions maintained at IITA has not been conducted. CoBB severity was evaluated in 103 CLR accessions from five African countries, the US, The Philippines, and Sri Lanka by artificially inoculating a highly virulent *Xav* strain in plants grown in a screenhouse. Highly significant (*P* < 0.0001) differences in susceptibilities to the disease were detected among the evaluated germplasm. Resistance was detected in several CLR accessions with two accessions from Nigeria and one from the US developing no disease symptoms. Our results indicate that several CLR accessions are valuable sources of resistance to CoBB and those could be used to breed for improved varieties with superior resistance to the disease. The resistant CLR accessions and others in IITA collection should be further investigated to identify additional beneficial traits that may contribute to the development of improved, commercially acceptable varieties.

## Highlights

• Highlights of Manuscript CROPRO-D-18-00414.
• Resistance to bacterial blight was detected in over 80% of the examined cowpea landrace accessions.
• Three accessions were classified as immune, developing no disease symptoms.
• Resistant accessions should be integrated into breeding programs to develop improved, resistant cultivars.

## 1 Introduction

Cowpea (*Vigna unguiculata* (L.) Walp.) is the most important legume native to Africa where is grown in the drier Savannas and Sahelian regions of sub-Saharan Africa (SSA). Those regions contribute circa 70% of global cowpea production (Boukar et al., 2012). Cowpea is also widely grown in certain nations of Latin America and Southeast Asia, and in the Southern US (FAO, 2016; Muchero et al., 2009). Across the world, over 12 million ha are cropped to cowpea with an annual grain production of >6.9 million tons. The largest cowpea producers are Nigeria, Niger, and Brazil (FAO, 2016). Grains, leaves, and haulms of cowpea are valued for their nutritious content for humans and livestock. Grains are rich in protein—generally composed of 25% protein—and both macro and micronutrients; leaves and haulms also contain valuable nutrients and are used primarily as fodder for livestock (Singh, 2006). In SSA, cultivation of cowpea provides social and economic benefits (e.g. market access, registration of cooperatives, cash for social functions) to smallholder farmers due to its many uses (Kristjanson et al., 2005; Langyintuo et al., 2003; Langyintuo and Lowenberg-DeBoer, 2006).

Although cowpea is well adapted to most regions in SSA, the crop is threatened by several pests and diseases, including cowpea bacterial blight (CoBB), caused by *Xanthomonas axonopodis* pv. *vignicola* (*Xav*). The first report of the disease was done in the US during the mid-20th century (Nandini, 2012). In Africa, it was reported in 1964 in Tanzania (Allen, 1981) while in Nigeria it was first reported in 1975 (Williams, 1975). To date, CoBB has been reported in most nations where cowpea is grown (Bastas and Sahin, 2017; Moretti et al., 2007; Nandini and Kulkarni, 2016; Shi et al., 2016). The major impact of *Xav* infection is on the leaves and depending on the susceptibility of the genotype it can cause complete defoliation (Claudius-Cole et al., 2014). Pods, seeds, and stems are also affected.

Strategies to control CoBB include seed treatment with bactericides, intercropping, use of pathogen-free seeds, and use of resistant germplasm (Sikirou and Wydra, 2004). Use of chemicals may be too expensive for smallholder farmers and/or may not be readily available for them (Shi et al., 2016). Use of cowpea germplasm with resistance to CoBB is a promising strategy with the potential to control the disease in an economic and sustainable manner (Emechebe and Lagoke, 2002). Sources of resistance to various diseases are often found in landraces or wild relatives of crops (Hegde and Mishra, 2009). Across SSA, most cowpea growers rely on self-saved seeds of cowpea landrace (CLR) accessions that have been grown in traditional agro-ecosystems over hundreds of generations (Uguru, 1998). CLR accessions may harbour resistance to *Xav*, because of exposure to the pathogen over centuries and long-term selection of resistant accessions by farmers.

Seed is the primary inoculum source of *Xav*. Planting seeds infected with *Xav* can result in either pre- or post-emergence seedling infection and subsequent mortality (Ganiyu et al., 2017). CLR heirloom seeds are typically not tested for the presence of *Xav* or any other pathogen. Therefore, risks of CoBB outbreaks are prevalent. In addition, farmers using CLR seeds not infected with *Xav* may have their crops infected due to *Xav* infecting cowpea and/or alternative hosts in neighbouring fields (Sikirou and Wydra, 2004).

IITA holds the world's largest and most diverse cowpea collection, with over 15,000 unique accessions from 88 countries representing 70% of African cultivars and nearly half the global diversity (Boukar et al., 2012). Only a small fraction of the collection has been evaluated for resistance to CoBB. It would, therefore, be valuable to screen CLR accessions with the aim of identifying sources of resistance for either continuous usage as accessions with known resistance to CoBB or to be integrated into breeding programs for development of resistant, improved cowpea varieties. Based on these considerations, 103 CLR accessions were evaluated for resistance to CoBB under screenhouse conditions in the current study. The knowledge obtained from the current study will aid to detect valuable CLR accessions possessing high levels of resistance to CoBB that could be integrated into breeding programs to develop cultivars with resistance to CoBB and other desirable traits.

## 2 Materials and methods

A total of 103 CLR accessions were evaluated for resistance to CoBB. The accessions, maintained at the IITA Gene Bank, were selected from a previous study examining the genetic diversity of CLR accessions for agromorphological descriptors conducted in IITA-Ibadan (M. Gedil et al., unpublished); the selected accessions are representative of the great diversity of the germplasm maintained at IITA. Accessions from Nigeria (52), South Africa (20), Tanzania (10), the US (9), Senegal (4), Uganda (4), The Philippines (2), Sri Lanka (1), and one of unknown origin, were used in the current study (Table 1). Two additional genotypes, Danila and IT84S-2246-4, which have been classified as resistant and susceptible to CoBB (Agbicodo et al., 2010), respectively, were provided by the IITA Cowpea Breeding Unit and were used as both positive and negative control treatments.

**Table 1:** Severity of cowpea bacterial blight among cowpea landrace (CLR) accessions and two advanced cultivars inoculated with a highly virulent strain of *Xanthomonas axonopodis* pv. *vignicola*.

Cowpea accession	Country of origin	Days to first disease symptom range^a^	Disease severity 22 dai^b^	Disease reaction^c^
TVu 58, TVu 64	Nigeria	–	0.00 k	I
TVu 102	USA	–	0.00 k	I
TVu 10, TVu 42, Danila	Nigeria	4–22	0.08 j-k	R
TVu 101	Tanzania	4	0.08 j-k	R
TVu 97	South Africa	12	0.08 j-k	R
TVu 41, TVu 52	Nigeria	4–22	0.10 i-k	R
TVu 80, TVu 84, TVu 96	South Africa	4–12	0.10 i-k	R
TVu 4, TVu 11, TVu 13, TVu 51, TVu 56, TVu 60, TVu 63	Nigeria	4–22	0.15 h-k	R
TVu 37, TVu 91	South Africa	4–12	0.15 h-k	R
TVu 70,	Senegal	4	0.16 h-k	R
TVu 54, TVu 73, TVu 76	Nigeria	4–7	0.20 h-k	R
TVu 87	Tanzania	22	0.20 h-k	R
TVu 71	Senegal	4	0.20 h-k	R
TVu 81, TVu 92	South Africa	4–7	0.20 h-k	R
TVu 19, TVu 50	Nigeria	4–12	0.25 h-k	R
TVu 69	Senegal	4	0.25 h-k	R
TVu 85	South Africa	4	0.27 h-k	R
TVu 88	Uganda	4	0.30 h-k	R
TVu 2, TVu 49	Nigeria	4–12	0.33 h-k	R
TVu 32, TVu 90	South Africa	4–7	0.33 h-k	R
TVu 38	Tanzania	7	0.33 h-k	R
TVu 98	Sri Lanka	4	0.33 h-k	R
TVu 6, TVu 44, TVu 55, TVu 59	Nigeria	4–7	0.38 h-k	R
TVu 78, TVu 95	South Africa	7–15	0.40 h-k	R
TVu 35	Unknown	4	0.42 h-k	R
TVu 99	Tanzania	12	0.42 h-k	R
TVu 18, TVu 43, TVu 47, TVu 67, TVu 75	Nigeria	4–12	0.50 h-j	R
TVu 26	USA	12	0.50 h-j	R
TVu 89	Uganda	4	0.50 h-j	R
TVu 39	Tanzania	12	0.55 g-j	R
TVu 86, TVu 79	Tanzania	4–7	0.58 g-i	R
TVu 8, TVu 9, TVu 14, TVu 33, TVu 45	Nigeria	4–12	0.58 g-i	R
TVu 104	Tanzania	4	0.63 f-i	R
TVu 77, TVu 82	South Africa	4–7	0.65 f-h	R
TVu 22	The Philippines	4	0.67 f-h	R
TVu 24	USA	4	0.67 f-h	R
TVu 57, TVu 62, TVu 65, TVu 74	Nigeria	4	0.67 f-h	R
TVu 34	Uganda	4	0.70 e-h	R
TVu 5, TVu 15, TVu 20	Nigeria	4–12	0.75 e-h	R
TVu 12, TVu 48	Nigeria	4–12	0.83 d-h	R
TVu 93	South Africa	7	0.83 d-h	R
TVu 7, TVu 53, TVu 68	Nigeria	4–12	0.86 c-h	R
TVu 31	Uganda	7	0.91 c-h	R
TVu 25, TVu 29	USA	12	0.92 c-h	R
TVu 36	South Africa	4	0.92 c-h	R
TVu 100, TVu 103	Tanzania	4–7	1.00 b-g	MS
TVu 16, TVu 17	Nigeria	4–12	1.00 b-h	MS
TVu 27	USA	4	1.00 b-h	MS
TVu 40, TVu 94	South Africa	4	1.00 b-h	MS
TVu 21	The Philippines	4	1.08 b-f	MS
TVu 72	Senegal	4	1.08 b-f	MS
TVu 3	Nigeria	7	1.13 b-f	MS
TVu 61	Nigeria	7	1.16 b-e	MS
TVu 83	South Africa	7	1.16 b-e	MS
TVu 1	Nigeria	12	1.25 b-d	MS
TVu 28	USA	4	1.25 b-d	MS
TVu 23	USA	4	1.33 b-c	MS
IT84S-2246-4	Nigeria	4	1.41 b	MS
TVu 30	USA	7	2.00 a	S
TVu 46	Nigeria	4	2.00 a	S
Danila water-inoculated	Nigeria	–	0.00 k	Resistant Control
IT84S-2246-4 water-inoculated	Nigeria	12	0.16 h-k	Susceptible Control

a Days after inoculation (dai) in which symptoms appeared. ‘-’ indicates that plants of those accessions did not develop disease symptoms in any of the two tests, at 22 dai.

b Disease severity values at the end of evaluations, 22 dai. Values are means of six replicates. Each replicate was composed of three plants. Means were separated using Tukey’s HSD test.

c Accessions were classified as immune (I), resistant (R), moderately susceptible (MS), and susceptible (S) based on severity values. Water inoculated accessions served as negative control treatments. Accessions with the same severity values and originating from the same country are grouped in the same row.

A *Xav* isolate obtained from a diseased cowpea plant grown in IITA experimental station at Minjibir, Kano State, Nigeria (12°10′42.0″N; 8°39′33.1″E), was used in the current study. The isolate, hereafter referred to as *Xav*-Minjibir, was highly pathogenic to diverse cowpea germplasm in studies conducted in our laboratory (unpublished). For inoculum preparation, *Xav*-Minjibir was grown on nutrient glucose medium (NG; 28 g l^−1^ Nutrient Agar, 20 g l^−1^ glucose) for 48 h at 28 °C. Inoculum suspensions were prepared by harvesting bacterial cells into sterilized deionized distilled water. Suspensions were adjusted turbidimetrically using a spectrophotometer to an optical density of 600 nm (0.3), or approximately 2.4 × 10^8^ colony forming units (CFU) ml^−1^.

CLR accessions were evaluated in their resistance to CoBB in a screenhouse at IITA-Ibadan (07°30′20.7″ N; 03°54′08.4″ E). Plastic pots of 20.3 cm in both diameter and height were disinfested with hot water, cleaned and filled with sun-dried, sterilized top loamy soil. Seeds were sown at 2.5 cm in depth on August 23 and October 24, 2016, for the first and second test, respectively. Three seeds per pot were planted and these were irrigated with tap water using a watering can every three days. The experiment was conducted twice over a two month period.

The experiments were arranged in a completely randomized design with three replications (one pot per replicate; each pot containing three plants) per accession. Six hours prior to the first inoculation and until completion of the evaluations, plants were misted with tap water from 10 a.m. to 4 p.m. using a fogging machine (Reldair Fogging System; Reldairbv, Edisonstraat, The Netherlands) to create a favourable environment for disease development. All three cowpea plants in a pot were inoculated twice on the first trifoliate leaves as described by Agbicodo et al. (2010). For the first and second tests, plants were inoculated 16 and 24 days after planting (dap). Approximately 3 ml of inoculum suspension was atomized on the lower surface of young expanding leaves using a Fisherbrand™ liquid sprayer (Fisher Scientific, Pittsburgh, PA). In the screenhouse, the average temperature was 24.0 °C and 25.5 °C for the first and second experiment, respectively. The average relative humidity in the screenhouse for both the first and second experiment was 88%. After inoculation, plants were covered with polyethene bags for 24 h to increase humidity in plant canopy, which is helpful to establishing infection (Agbicodo et al., 2010). In each test, an additional set of Danila and IT84S-2246-4 were inoculated with sterile, distilled water and served as the negative control treatment.

Prior to inoculation, CoBB symptoms were not detected in any plant. After inoculation, all plants in each pot were inspected for CoBB symptoms throughout three weeks. Disease severity was rated on a scale of 0–4 as described by Agbicodo et al. (2010), where 0 = no visible symptoms, 1 = leaf spots covering <10% leaf area, 2 = blight affecting 10–50% leaf area, 3 = severe blight on >50% leaf area, and 4 = inoculated trifoliate leaf shed; sometimes blight in non-inoculated leaves. Disease symptoms were assessed at 4, 7, 12, 15, 19, and 22 days after inoculation (dai). CoBB severity index was calculated by averaging disease values from all three plants of each accession. In each experiment, variances within means of the cowpea accessions were homogeneous. This allowed to pool the data from the two experiments for statistical analyses. Analysis of variance (ANOVA) models were conducted in SAS version 9.0 (SAS Institute, Cary, NC) using score values converted to the respective percentages. Means were separated using Tukey's HSD test (α** = **0.05).

## 3 Results

There were significant (*P* < 0.05) differences in CoBB disease severity indices among the examined cowpea germplasm. At 22 dai, averages of disease severity values ranged from 0.0 (no detectable symptoms) to 2.0 (Table 1). Typical symptoms appeared in the form of small, water-soaked, brown lesions, which gradually expanded and coalesced to form large necrotic lesions. Symptoms were visible in some CLR accessions as early as 4 dai (Table 1). Based on disease severity values at the end of evaluations (22 dai), accessions were classified as immune, resistant, moderately susceptible, and susceptible to CoBB.

The susceptible group, with an average disease severity of 2.0, consisted of accessions TVu 30 and TVu 46, from the US and Nigeria, respectively (1.9% of evaluated germplasm; severity index = 2.0). The moderately susceptible group included 15 CLR accessions and the susceptible control IT84S-2246-4 (15.1% of evaluated germplasm; severity index range = 1.0–1.4). The resistant group was the largest and includes 83 CLR accessions and resistant control Danila (79.2% of evaluated germplasm; severity index range = 0.1–0.9). Remarkably, three CLR accessions, TVu 58 and TVu 64, from Nigeria, and TVu 102, from the US, exhibited no disease symptoms (2.9% of evaluated germplasm; severity index of 0.0, immune group). Water-inoculated Danila plants had no disease symptoms while water-inoculated IT84S-2246-6 plants had a disease severity index of 0.2 with symptoms appearing at 12 dai. The appearance of CoBB symptoms in water-inoculated IT84S-2246-6 suggests that seeds were contaminated with *Xav*, perhaps by an isolate with low virulence, although this was not tested.

Several resistant accessions had uniformly low disease severity values in both tests. Minor lesions occurred in accessions with disease severity indices of 0.1 and 0.2 (Table 1); TVu 41 and TVu 87 developed CoBB symptoms only at 22 dai. In general, for both susceptible and moderately susceptible accessions, CoBB severity progressed after 7 dai. CoBB reached its stationary phase at 19 dai in accessions within these categories and there were no significant (*P* > 0.05) differences in disease severity indices between the last two observation periods (data not shown).

## 4 Discussion

From the great diversity of cowpea germplasm maintained at IITA Gene Bank (Boukar et al., 2012), improved genotypes have been screened for resistance to CoBB and minor emphasis has been given to CLR accessions (Agbicodo et al., 2010; Sikirou et al., 2001). Knowledge of resistance of germplasm to important diseases is a valuable resource for plant breeding programmes because it helps to identify accessions in which to obtain genes associated with resistance. The current study evaluated variation in resistance to CoBB among 103 CLR accessions when challenged with a virulent strain of *Xav*. The evaluated accessions are maintained at IITA Gene Bank and constitute only a fraction of cowpea's diversity. The large majority of the examined CLR accessions (79.8%) possessed resistance to CoBB. Three accessions (2.8%) did not express disease symptoms in any of the two tests. These CLR accessions are sources of resistance that should be considered for inclusion into breeding programs to develop a pipeline of inbred lines with high resistance to CoBB through conventional breeding and/or marker-assisted selection.

Although seed is the primary inoculum source of *Xav* (Ganiyu et al., 2017), our evaluations aimed to detect resistance to CoBB by directly inoculating leaves. Apart from the seed, *Xav* can overwinter on crop residues, fall-sown cereals, and perennial grasses and infection may occur after spread of bacterial ooze from diseased plants by raindrops, plant to plant contact, and insect transmission (Moretti et al., 2007; Sikirou and Wydra, 2004; Zandjanakou-Tachin et al., 2007). Inoculation methods used to screen for resistance to CoBB include seed inoculation, soil inoculation, stem injection, and foliar spraying (Kutama et al., 2013; Sikirou et al., 2001). The later was used in our study because it allows to inoculate leaves directly, without damage, and high disease pressure is provided.

Variability in disease symptom expression among the examined germplasm was detected (Table 1). Disease symptoms appeared as conspicuous yellow halos and chlorotic borders around necrotic lesions, which are typical of CoBB (Okechukwu et al., 2010). Some accessions exhibited symptoms at 4 dai while others had no symptoms even after 22 dai; CLR accessions (TVu 58, TVu 64, and TVu 102) were completely resistant to CoBB. Leaf dropping occurred in some plants of most of the moderately susceptible and susceptible accessions. No trend was detected in which susceptibility categories were influenced by geographic origin of CLR accessions (Table 1). Our results identified several CLR accessions with superior genetic backgrounds that could lead to the identification of genes, quantitative trait loci (QTL), and/or single nucleotide polymorphisms (SNP) associated with resistance to CoBB. Future work evaluating resistance to CoBB in other sets of germplasm should consider including TVu 102, TVu 64, and TVu 58 as resistant accessions and TVu 46 and TVu 30 as susceptible accessions.

CLR accessions TVu 102, TVu 64, and TVu 58 should be integrated into breeding programs to develop cowpea improved germplasm. Indeed, the three accessions had greater resistance than resistant control Danila, a cultivar used in CoBB screening studies and development of markers associated with resistance to CoBB (Agbicodo et al., 2010). In the study conducted by Agbicodo et al. (2010) even the most resistant genotype, IT81D-1228-14, exhibited disease symptoms. However, in that study, two different *Xav* genotypes, *Xav*18 and *Xav*19, were used and it should be investigated whether the immunity observed in TVu 102, TVu 64, and TVu 58 against *Xav*-Minjibir will hold against *Xav*18, *Xav*19, or other *Xav* genotypes. Uniformly low disease scores (i.e., with severity index of 0.1 and 0.2) were detected in 23 CLR accessions (Table 1). Those accessions should also receive consideration for integration into breeding programs, especially if possessing desirable agronomic traits. Future research efforts should investigate similarities in both resistance mechanisms and inheritance of resistance among the immune and resistant CLR accessions detected in the current study.

## 5 Conclusion

Millions of people across SSA and other regions rely on cowpea as a primary source of both food and income. Use of both immune and resistant CLR accessions identified in the current study should be promoted among cowpea growers to rapidly reduce incidences of CoBB. In addition, immune and resistant accessions should be integrated into traditional and/or molecular assisted breeding programs for the development of cultivars that can resist high pressures of *Xav* across SSA and elsewhere. Improved materials with resistance to CoBB would reduce losses associated with the disease. Landraces provide valuable sources of variation for beneficial traits, including disease resistance (Smýkal et al., 2015). Efforts should be geared to exploit landrace accessions to both prevent their disappearance and aid in crop improvement. It is common for landraces to out-perform modern cultivars when challenged with both biotic and abiotic stresses (Dwivedi et al., 2016). Here we provide evidence that CLR accessions are valuable materials in which to identify traits of importance for integration into breeding programs, such as resistance to major diseases. Both durable and multiple-disease resistance can be found in crop landraces and/or their wild relatives (Tanksley and McCouch, 1997; Wiesner-Hanks and Nelson, 2016). Only a small fraction of IITA CLR collection was tested for resistance to CoBB. Future research efforts should expand the number of accessions screened for resistance to CoBB and other pathogens, among other beneficial traits.

## Conflicts of interest

Authors declare no conflict of interest. All authors consent to the submission of this manuscript.
